# Intermolecular functional coupling between phosphoinositides and the potassium channel KcsA

**DOI:** 10.1016/j.jbc.2022.102257

**Published:** 2022-07-15

**Authors:** Takunari Kiya, Kohei Takeshita, Akira Kawanabe, Yuichiro Fujiwara

**Affiliations:** 1 Laboratory of Molecular Physiology & Biophysics, Faculty of Medicine, Kagawa University, Miki-cho, Kagawa, Japan; 2 RIKEN SPring-8 Center, Sayo-cho, Hyogo, Japan

**Keywords:** potassium channel, gating, phosphoinositides, protein–lipid interaction, electrophysiology, bilayer recording, CBB, contact bubble bilayer, KcsA, the K^+^ channel of *Streptomyces lividans*, K_d_, dissociation constant, Kir, inward rectifier K^+^ channel, MST, microscale thermophoresis, PI, phosphatidylinositol, PI(3,4,5)P_3_, phosphatidylinositol 3,4,5-trisphosphate, PI(3,4)P_2_, phosphatidylinositol 3,4-bisphosphate, PI(4,5)P_2_, phosphatidylinositol 4,5-bisphosphate, PI(4)P, phosphatidylinositol 4-phosphate, PIPns, phosphoinositides, Po, open probability, POPC, 1-palmitoyl-2-oleoylphosphatidylcholine, POPG, 1-palmitoyl-2-oleoylphosphatidylglycerol, PIPn, phosphoinositide, DDM, n-dodecyl-β-D-maltoside

## Abstract

Biological membranes are composed of a wide variety of lipids. Phosphoinositides (PIPns) in the membrane inner leaflet only account for a small percentage of the total membrane lipids but modulate the functions of various membrane proteins, including ion channels, which play important roles in cell signaling. KcsA, a prototypical K^+^ channel that is small, simple, and easy to handle, has been broadly examined regarding its crystallography, *in silico* molecular analysis, and electrophysiology. It has been reported that KcsA activity is regulated by membrane phospholipids, such as phosphatidylglycerol. However, there has been no quantitative analysis of the correlation between direct lipid binding and the functional modification of KcsA, and it is unknown whether PIPns modulate KcsA function. Here, using contact bubble bilayer recording, we observed that the open probability of KcsA increased significantly (from about 10% to 90%) when the membrane inner leaflet contained only a small percentage of PIPns. In addition, we found an increase in the electrophysiological activity of KcsA correlated with a larger number of negative charges on PIPns. We further analyzed the affinity of the direct interaction between PIPns and KcsA using microscale thermophoresis and observed a strong correlation between direct lipid binding and the functional modification of KcsA. In conclusion, our approach was able to reconstruct the direct modification of KcsA by PIPns, and we propose that it can also be applied to elucidate the mechanism of modification of other ion channels by PIPns.

Phosphoinositides (PIPns) are membrane lipids in the cytoplasmic leaflet of the plasma membrane where they coexist with a variety of membrane proteins, including receptors and ion channels. PIPns are negatively charged lipids and are present in only a few percent of mammalian biological membranes (1, 2, 3). The level of PIPns in the plasma membrane is dynamically modulated by phosphatases, kinases, and phospholipases (4). These lipid signals play important roles in various aspects of cell biology, including endosome dynamics (5), cell adhesion (6), and oncogenesis (7). In addition, PIPns have been shown to exert a modulatory effect on the activity of various ion channels (Fig. 1*A*) (8, 9, 10). For example, PI(4,5)P_2_ directly activates the inward rectifier K^+^ channel (Kir) (11, 12, 13), and insufficient interaction between PI(4,5)P_2_ and Kir channels leads to channelopathies (8, 14). PIPns also modulate the activity of several channels including two pore domain K^+^ channels (15), voltage-gated K^+^ channels (16, 17, 18), calcium-activated K^+^ channels (19), transient receptor potential channels (20), hyperpolarization-activated and cyclic nucleotide–gated channels (21, 22), epithelial Na^+^ channels (23), ATP receptor (P2X) channels (24) and variety of transporters (9). As with other membrane proteins, there are two possible modes of ion channel modification by PIPns (9). Structural biology has shown that some ion channels have binding pockets tailored to PIPns and their activities are regulated by PIPns binding to the pocket (25, 26), while other channels do not have clear binding pockets and their activities are regulated through electrostatic interactions with PIPns (24, 27). Although experiments with mutants have been widely conducted to analyze the functional modification by PIPns, to the best of our knowledge there has been no direct physicochemical analysis of the molecular interactions between PIPns and ion channel proteins.

**Figure 1:**
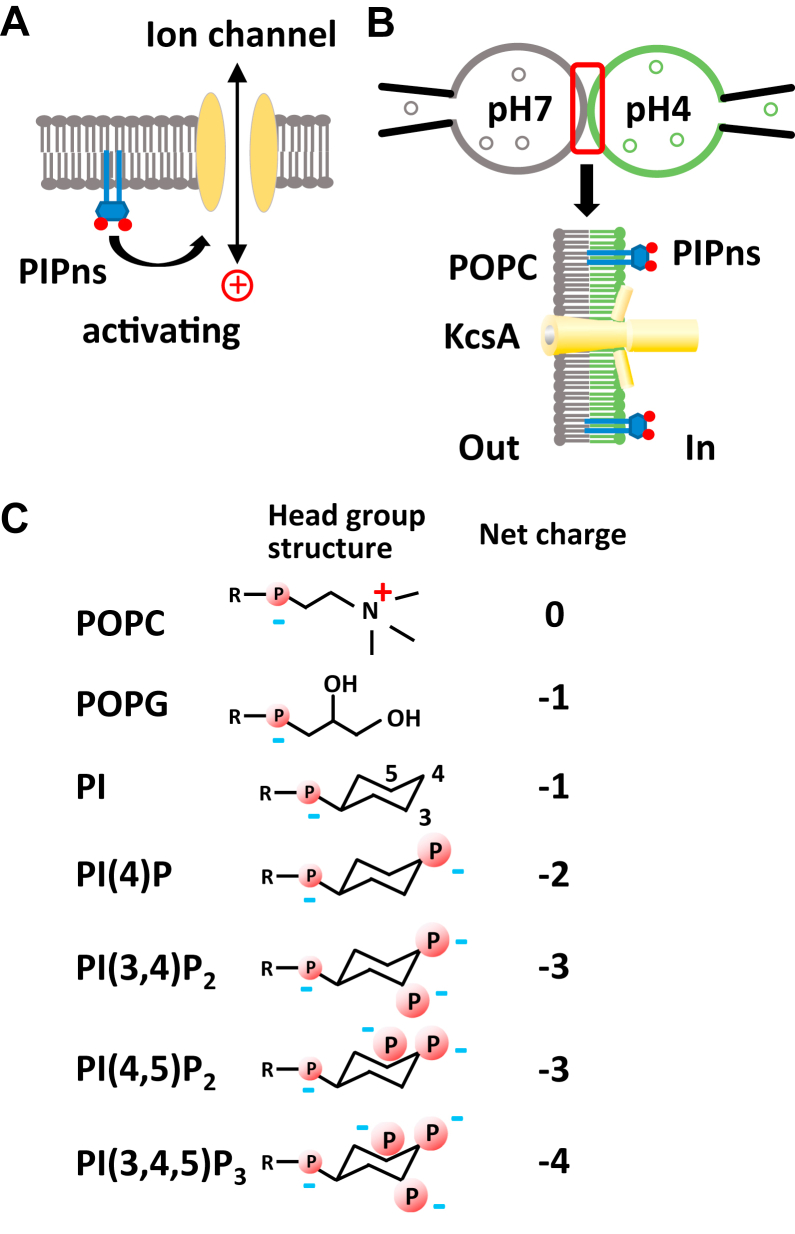
**Schematic drawings of the PIPns physiology and contact bubble bilayer analysis.***A*, the level of PIPns is dynamically modulated by cellular enzymes, and some PIPns bind to ion channels and modify their activity. *B*, the configuration of contact bubble bilayer recording. KcsA, which is opened by intracellular acidification (pH 4.0), was recorded by mixing PIPns with inner leaflets of lipid bilayers. *C*, lipid headgroup structures used in this study. P indicates negatively charged phosphate groups. R indicates general acyl chain backbones. PI, phosphatidylinositol; PI(4)P, phosphatidylinositol 4-phosphate; PI(3,4,5)P_3_, phosphatidylinositol 3,4,5-trisphosphate; PI(3,4)P_2_, phosphatidylinositol 3,4-bisphosphate; PI(4,5)P_2_, phosphatidylinositol 4,5-bisphosphate; PIPn, phosphoinositide; POPC, 1-palmitoyl-2-oleoylphosphatidylcholine; POPG, 1-palmitoyl-2-oleoylphosphatidylglycerol.

Since mammalian ion channels are large and complex structures, we used the small, simple, and easy-to-handle KcsA in this study to analyze ion channel modification by PIPns. KcsA is the K^+^ channel of *Streptomyces lividans* that has been intensively studied both functionally and structurally (28, 29, 30, 31). Although small and simple, it shows gating and shares its molecular structure with larger and more complex mammalian ion channels, such as an ion selectivity filter, and it is therefore being studied as a prototypical ion channel. KcsA gates under acidic pH, and it is also known to be activated by lipid modification and membrane stretch (32, 33, 34). Lipid-bound conformations have been reported in crystal structures, and lipid interaction sites that modify the function of KcsA have been predicted by mutation studies (35, 36, 37). In particular, it has been reported that KcsA opens when the cytoplasmic leaflet side of the lipid bilayer is formed with negatively charged phospholipids such as 1-palmitoyl-2-oleoylphosphatidylglycerol (POPG), and a sensor function for anionic phospholipids has also been proposed (36, 38). However, due to the lack of methodology to analyze the direct binding of lipids to ion channel membrane proteins, quantitative analysis has not been sufficiently carried out. It is also worth investigating research concerns such as the concentration at which the ion channel function modifies and if the functional regulation of ion channels occurs through the direct binding of lipid molecules to ion channels. These are important questions to be addressed, based on the properties of KcsA, a prototypic ion channel, when considering the mechanism of functional regulation of ion channels by PIPns. In the present study, we used electrophysiological and molecular interaction analysis of KcsA to elucidate the functional coupling between phospholipids (Fig. 1*C*) and ion channels.

## Results

We first investigated whether PIPns can functionally modify KcsA using lipid bilayers, a pure reconstituted system in which only lipids and channels are present. The contact bubble bilayer (CBB) method, which is a new method for lipid bilayer analysis, has developed in recent years (39, 40). Using this method, it is easy to control the lipid composition, and the measurement technique can be adapted from the patch clamp technique (39, 40) (Fig. 1*B*). It is much easier to prepare asymmetric membranes using CBB than the conventional planar bilayer method. We prepared an outer lipid membrane bubble using 1-palmitoyl-2-oleoylphosphatidylcholine (POPC) proteoliposomes containing purified KcsA E71A protein in a pH 7.0 recording solution, and an inner lipid membrane bubble using POPC liposomes containing phospholipids such as PIPns in a weight % concentration in a pH 4.0 recording solution (Fig. 1*B*). The two bubbles were attached to form a CBB bilayer. This configuration allowed analysis of the electrophysiological properties of KcsA gating under intracellular acidic pH conditions and the functional modification of the intracellular membrane by PIPns (Fig. 1*B*). To quantitatively analyze the modification by PIPns, we used the E71A mutant because it has a stable and high channel open probability due to the lack of channel inactivation (41) while retaining other native channel characteristics (42). The E71A mutant, whose structure has also been analyzed and retains the basic skeleton of the KcsA wildtype (WT) except for structural differences in the selectivity filter (41, 43), has served as an alternative channel in the analysis of many fundamental properties of KcsA, including pH-dependent gating, lipid-mediated gating, and the effects of inhibitors (36, 44, 45, 46), and also in membrane reconstruction experiments using novel methods (47, 48).

**Figure 2:**
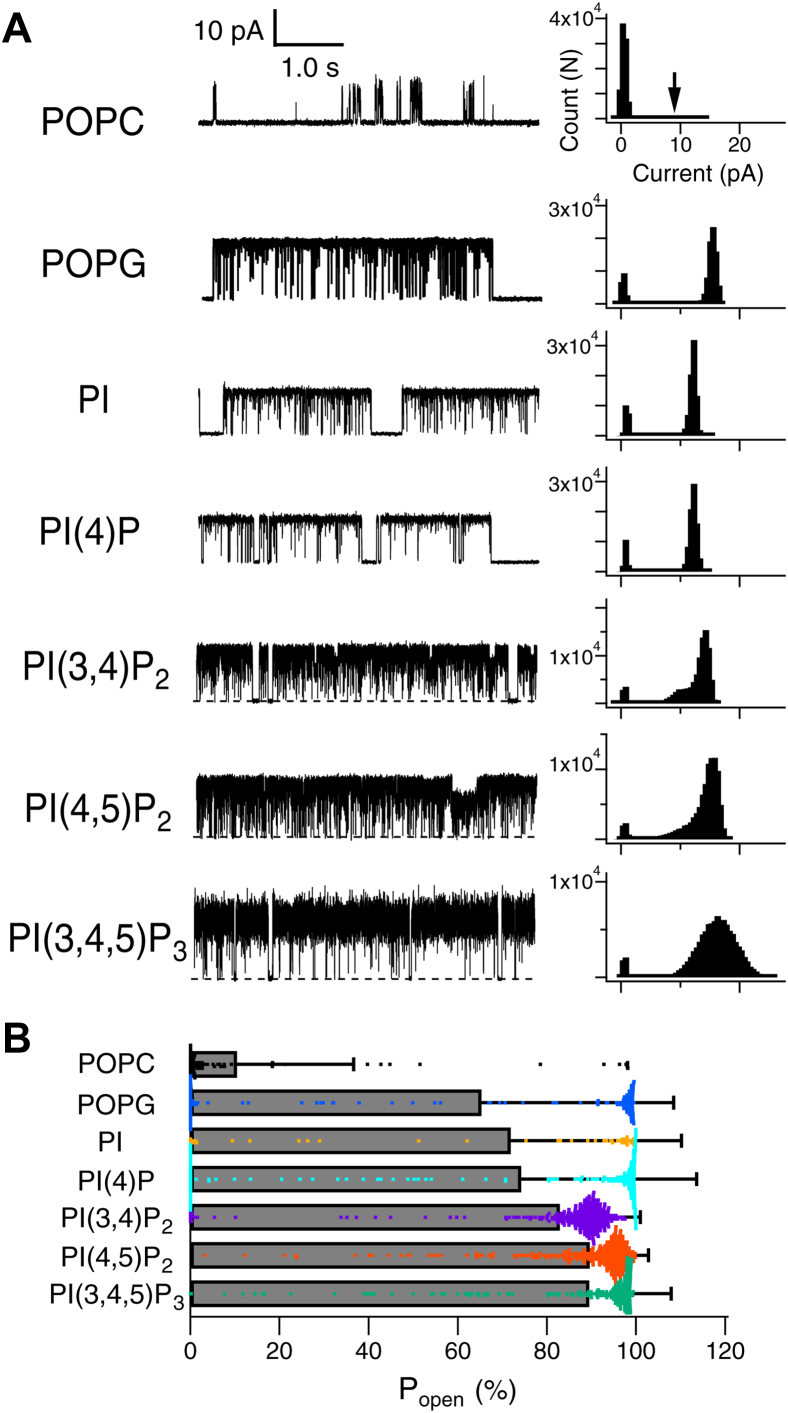
**Single-channel recording with increased open probability due to PIPns.***A*, representative current traces of the single-channel recordings of KcsA E71A at 100 mV in the presence of phospholipids mixed into the lipid bilayer. The *top row* is a recording from a 100% POPC membrane; the other recordings are from a 10% mixture of the indicated phospholipids (*e.g.*, POPG: 90% POPC + 10% POPG). Histograms of the amplitude are shown on the right. An *arrow* indicates the open amplitude level in POPC. *B*, comparison of the open probability among the phospholipids added. Currents were recorded using the 100% POPC membrane or membranes containing 10% of other lipids. All recorded data are divided into segments of 1 s each for analysis, and the number of the data segments and recording trials were 116 segments/3 recordings in POPC; 145/3 in POPG; 48/3 in PI; 223/5 in PI(4)P; 368/4 in PI(3,4)P_2_; 390/6 in PI(4,5)P_2_; and 280/4 in PI(3,4,5)P_3_. All analyzed data of the open probability were plotted with *colored dots*, and bars with errors indicate means ± SD. PI, phosphatidylinositol; PI(4)P, phosphatidylinositol 4-phosphate; PI(3,4,5)P_3_, phosphatidylinositol 3,4,5-trisphosphate; PI(3,4)P_2_, phosphatidylinositol 3,4-bisphosphate; PI(4,5)P_2_, phosphatidylinositol 4,5-bisphosphate; POPC, 1-palmitoyl-2-oleoylphosphatidylcholine; POPG, 1-palmitoyl-2-oleoylphosphatidylglycerol.

Single-channel recordings measured in POPC membranes mixed with 10% phospholipids are shown in Figure 2. KcsA recorded from the control 100% POPC membrane showed a low probability of opening with very short bursts (Fig. 2*A*, top). The addition of 10% POPG increased the open probability (Po) (Fig. 2*A*), which is consistent with previous reports for 100% POPG under a planar bilayer (36). We found that the addition of PIPns increased Po significantly (*p* < 0.001 compared with POPC) (Fig. 2). Phosphatidylinositol 3,4-bisphosphate [PI(3,4)P_2_], phosphatidylinositol 4,5-bisphosphate [PI(4,5)P_2_], and phosphatidylinositol 3,4,5-trisphosphate [PI(3,4,5)P_3_] were more effective than phosphatidylinositol (PI), phosphatidylinositol 4-phosphate [PI(4)P], and POPG in increasing Po. The Po of PI(3,4)P_2_, PI(4,5)P_2_, and PI(3,4,5)P_3_ were significantly higher with each compared with the Po of PI, PI(4)P, and POPG, respectively (*p* < 0.001; however, the significant difference between PI(3,4)P_2_ and PI was *p* = 0.026). The effect of increasing the amplitude of the current was observed in POPG, PI(4,5)P_2_, and PI(3,4,5)P_3_ [POPG: 15 ± 2.6 pA (n = 3); PI(4,5)P_2_: 15 ± 3.5 pA (n = 3); PI(3,4,5)P_3_: 14 ± 3.2 pA (n = 4); *p* < 0.05 in all cases compared with POPC: 8.1 ± 3.8 pA (n = 6)], while no significant differences were found for other PIPns [PI: 11 ± 1.7 pA (n = 3), PI(4)P: 12 ± 1.0 pA (n = 5), and PI(3,4)P_2_: 11 ± 1.7 pA (n = 4)] (Fig. 2*A*).

**Figure 3:**
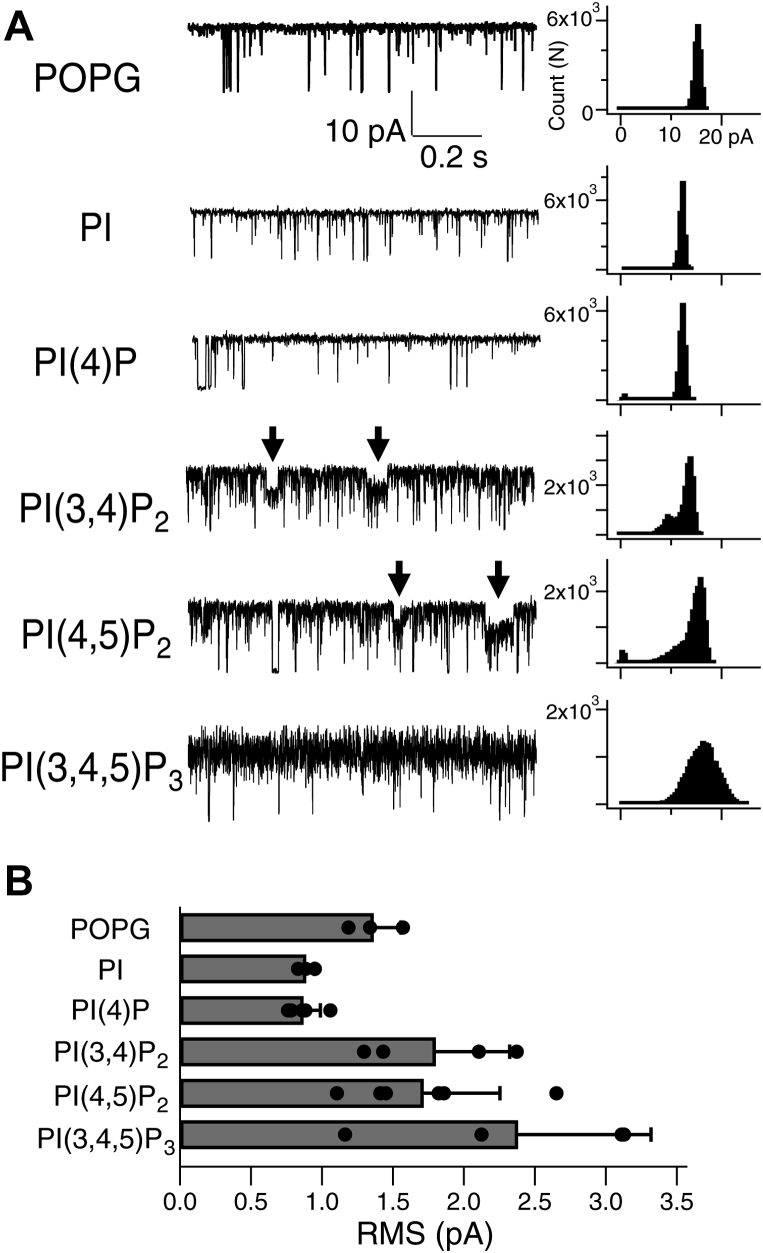
**Comparison of the open channel noise among the tested phospholipids.***A*, representative current traces of the single-channel recordings of KcsA E71A at 100 mV with the mixed phospholipids at 10% into the POPC lipid bilayer. *Arrows* indicate subconducting states. *B*, comparison of the open channel noise among phospholipids. All data of root-mean-squares (RMS) were plotted, and bars with errors indicate means ± SD (n = 3–6). PI, phosphatidylinositol; PI(4)P, phosphatidylinositol 4-phosphate; PI(3,4,5)P_3_, phosphatidylinositol 3,4,5-trisphosphate; PI(3,4)P_2_, phosphatidylinositol 3,4-bisphosphate; PI(4,5)P_2_, phosphatidylinositol 4,5-bisphosphate; POPC, 1-palmitoyl-2-oleoylphosphatidylcholine; POPG, 1-palmitoyl-2-oleoylphosphatidylglycerol.

We observed that the addition of phospholipids resulted in a difference in open channel noise. The zoomed-in current traces are shown in Figure 3*A*, and the root-mean-squares of the intraburst current amplitude are summarized in Figure 3*B*. The open channel noise of KcsA recorded from the PI(3,4)P_2_, PI(4,5)P_2_, and PI(3,4,5)P_3_ membranes was observed to be larger than that of the others. In addition, a clear subconducting state was observed in the current traces of PI(3,4)P_2_ and PI(4,5)P_2_ (arrows in Fig. 3*A*) but not in the current trace of PI(3,4,5)P_3_.

**Figure 4:**
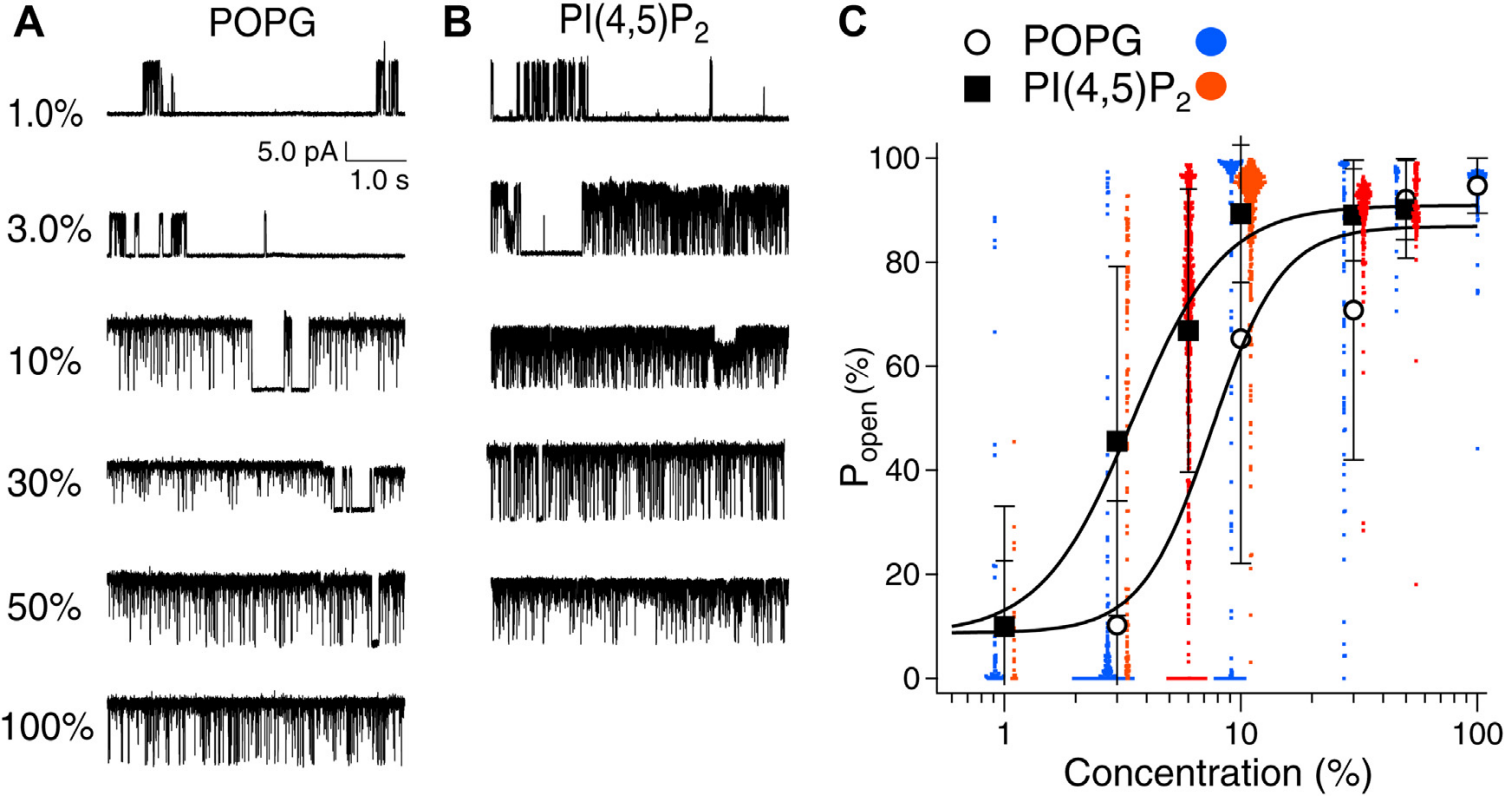
**Comparison of the dose–response relationship between POPG and PI(4,5)P**_**2**_**.***A* and *B*, representative current traces of single-channel recordings of KcsA E71A from POPC membranes mixed with the indicated concentrations of POPG or PI(4,5)P_2_. The current trace of PI(4,5)P_2_ 10% is the same trace as PI(4,5)P_2_ in Figure 2*A*. *C*, concentration-open probability relationship for POPG and PI(4,5)P_2_. All data of the open probability at each concentration were plotted as *blue*/*red dots*. Data are means ± SD. The number of the data segments and recording trials were 64 segments/3 recordings (1%), 154/3 (3%), 135/3 (10%), 74/3 (30%), 26/2 (50%), 181/3 (100%) in POPG; 20/2 (1%), 107/3 (3%), 533/5 (6%), 367/3 (10%), 169/2 (30%), 91/3 (50%) in PI(4,5)P_2_. The symbols used are as indicated in the figure. PI(4,5)P_2_, phosphatidylinositol 4,5-bisphosphate; POPC, 1-palmitoyl-2-oleoylphosphatidylcholine; POPG, 1-palmitoyl-2-oleoylphosphatidylglycerol.

In a previous report, KcsA E71A showed an increase in Po to approximately 0.9 when the cytoplasmic leaflet membrane was 100% POPG (36). However, since the effect of POPG at low content on Po had not been analyzed, the present study analyzed the effect of lipids at low content and examined the differences between lipids. In our recordings, we observed an increase in Po of approximately 0.7, even at 10% POPG, and a higher Po (approximately 0.9) was also observed for PI(3,4)P_2_, PI(4,5)P_2_, and PI(3,4,5)P_3_ than for POPG (Fig. 2). We hypothesized that this difference was due to differences in affinity, and we therefore analyzed the dose–response of phospholipids on the function of KcsA (Fig. 4). Single-channel recordings were performed under the conditions elucidated in Figure 2 using various concentrations of POPG or PI(4,5)P_2_ (Fig. 4, *A* and *B*). As the concentration of POPG and PI(4,5)P_2_ decreased, the Po of both POPG and PI(4,5)P_2_ decreased, approaching that of POPC (Po = 0.10 ± 0.26) (Fig. 4, *A* and *B*, top traces). At 3% POPG the Po hardly increased (Po = 0.10 ± 0.23 in POPG) (Fig. 4*A*), while a marked increase in the Po was observed at 3% PI(4,5)P_2_ (Po = 0.46 ± 0.33 in PI(4,5)P_2_; *p* < 0.001 compared with 100% POPC) (Fig. 4*B*). Concentration–Po relationships are shown in Figure 4*C*, where the dose–response curve for PI(4,5)P_2_, to the left of the POPG curve, suggests a strong effect on the KcsA opening (EC_50_: PI(4,5)P_2_, 3.6%; POPG, 7.5%). The molecular weight of PI(4,5)P_2_ was higher than that of POPG (POPC, 760.1; POPG, 771.0; and PI(4,5)P_2_, 1074.2), and the EC_50_ value was underestimated for PI(4,5)P_2_. The corrected EC_50_ value of PI(4,5)P_2_, calculated by converting the dose–response curve with the molecular weight of POPG, was 2.6%, which was approximately 2.9 times higher than that of POPG. There is a previous study reporting the dose–response of POPG content in bilayers on Po of KcsA wildtype (WT) (38), and the EC_50_ was reported to be higher than that found in the present study. This is probably due to differences in the channel (WT vs. E71A) and in membrane symmetry, with POPG present in the entire bilateral membrane or only in the unilateral membrane.

**Figure 5:**
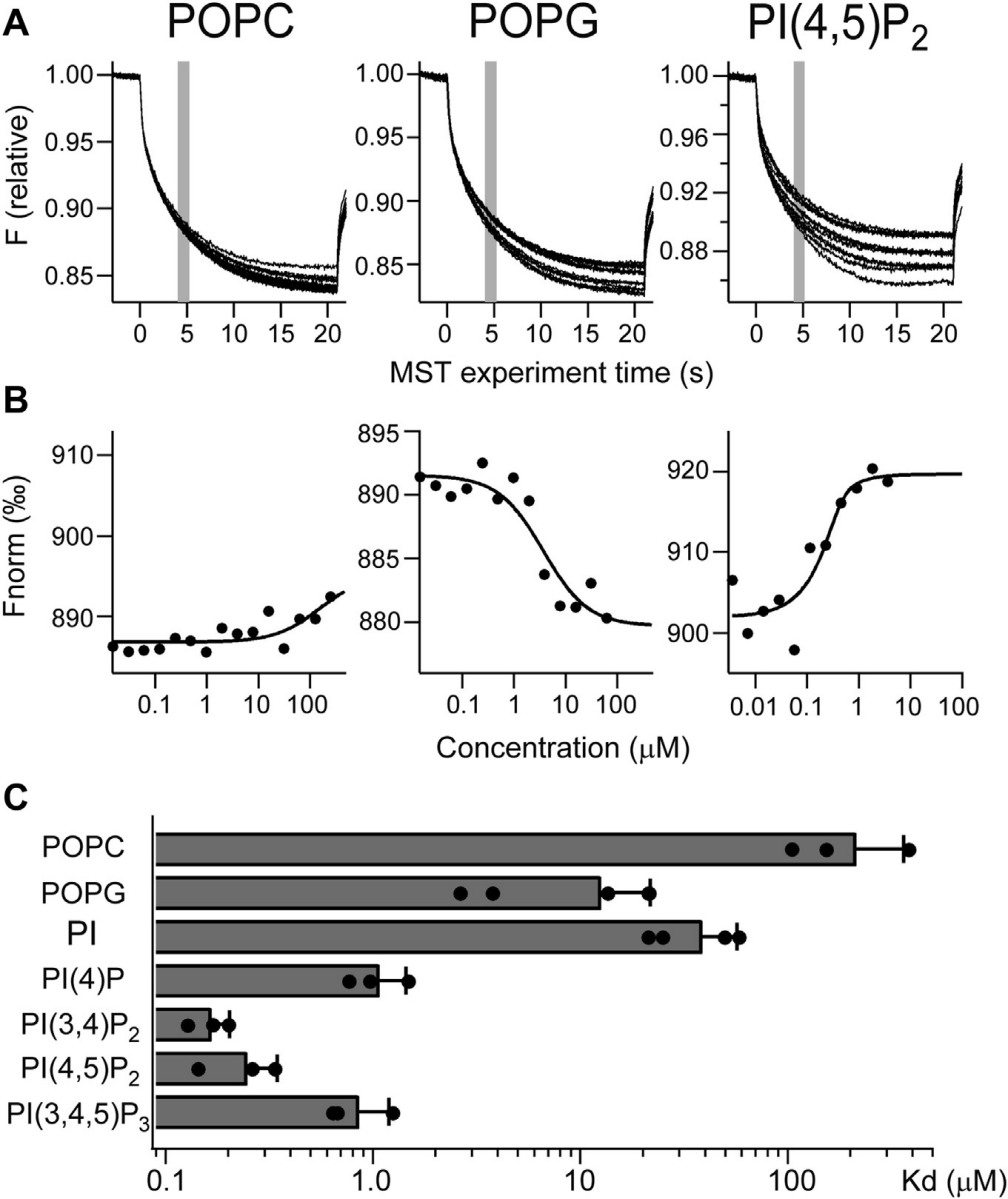
**KcsA–lipids interaction as elucidated by microscale thermophoresis analysis.***A* and *B*, the interaction of KcsA with phospholipids was monitored by fluorescent-molecular signals labeled on KcsA. The representative fluorescent signal traces of phospholipid titration are shown. Fittings of the change in fluorescence signal using Hill equation are also shown. *C*, comparison of K_d_ values among the phospholipids binding to KcsA. All data were plotted, and bars with errors indicate means ± SD (n = 3–5). PI, phosphatidylinositol; PI(4)P, phosphatidylinositol 4-phosphate; PI(3,4,5)P_3_, phosphatidylinositol 3,4,5-trisphosphate; PI(3,4)P_2_, phosphatidylinositol 3,4-bisphosphate; PI(4,5)P_2_, phosphatidylinositol 4,5-bisphosphate; POPC, 1-palmitoyl-2-oleoylphosphatidylcholine; POPG, 1-palmitoyl-2-oleoylphosphatidylglycerol.

**Figure 6:**
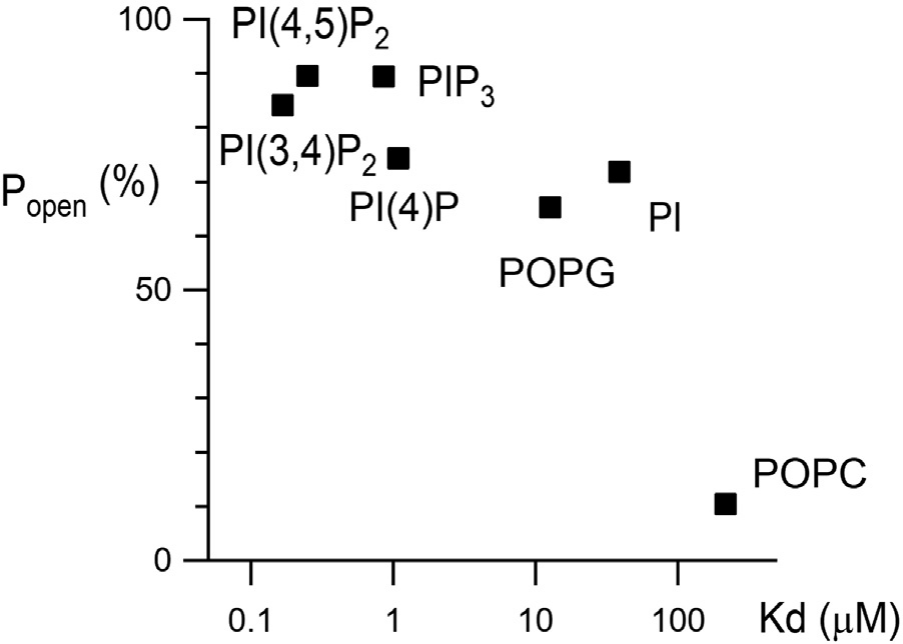
**Open probability *versus* binding affinity.** Correlations between the open probabilities under 10% lipid mixture (Fig. 2*B*) and the K_d_ values (Fig. 5*C*) were plotted. PI, phosphatidylinositol; PI(4)P, phosphatidylinositol 4-phosphate; PI(3,4)P_2_, phosphatidylinositol 3,4-bisphosphate; PI(4,5)P_2_, phosphatidylinositol 4,5-bisphosphate; PIP_3_, phosphatidylinositol 3,4,5-trisphosphate; POPC, 1-palmitoyl-2-oleoylphosphatidylcholine; POPG, 1-palmitoyl-2-oleoylphosphatidylglycerol.

It is worth investigating if the differential effect of phospholipids on KcsA opening is due to differences in binding affinity. There have been no reports on the direct analyses of the binding affinities of ion channels to lipids and their correlation with functional modifications. We used microscale thermophoresis (MST) analysis (49, 50) to estimate the binding affinity between KcsA and phospholipids. Phospholipid titration revealed changes in the MST signals from fluorescent molecules attached to the KcsA E71A protein (Fig. 5*A*). An increase in MST fluorescence was observed when PI(4,5)P_2_ was titrated, whereas a decrease was observed when POPG was titrated (Fig. 5*A*). Representative dose–response relationships of the MST signals were plotted (Fig. 5*B*), and the dissociation constants (K_d_) determined by fitting the accumulated data are summarized in Figure 5*C*. POPC showed the lowest affinity, and PI(3,4)P_2_ and PI(4,5)P_2_ showed the two with the highest affinity; the affinity of POPG was weaker than that of PI(4,5)P_2_ and comparable with that of PI (Fig. 5*C*). The K_d_ values of each phospholipid (PIPns and POPG) were significantly different from those of POPC. POPG and PI(4,5)P_2_, which showed difference in EC_50_ (Fig. 4), also showed statistically significant differences in K_d_ (*p* < 0.001). The K_d_ values of PI(4,5)P_2_ also showed significant difference from PI (*p* < 0.001), while they did not show significant difference from PI(4)P, PI(3,4)P_2_, and PI(3,4,5)P_3_. The Po values under 10% lipid mixture (Fig. 2*B*) were plotted against the K_d_ values, and the correlation coefficient was calculated to be -0.84 (Fig. 6). Overall, these results suggested a high correlation between PIPns binding and KcsA modification.

## Discussion

In the present study, we quantitatively analyzed the functional regulation and binding of PIPns to the prototypical ion channel KcsA to gain a general understanding of the modification mechanism by PIPns, which is responsible for lipid signaling in biological membranes and regulating the function of many ion channels. For the first time, we quantitatively demonstrated that phospholipids with stronger direct binding affinity lead to stronger functional modifications of KcsA.

Several previous studies have been conducted on the modification of KcsA by lipids. Anionic lipids, such as POPG, have been reported to markedly increase the Po of KcsA (32, 38). Negatively charged lipids in the cytoplasmic leaflet are reported to be a key factor in gating. From experiences with mutants, it has been proposed that negatively charged lipids interact with positively charged residues on the intracellular M0 helix, located just upstream of the first transmembrane helix (TM1), to stabilize the open structure (36). The present study, which focused on the lipid composition of the inner leaflet, may suggest a similar mechanism for the functional modification exerted by PIPns on KcsA. In addition, recent NMR-based binding study indicated that anionic lipids such as DOPG bound to the K^+^ selectivity filter located on the outer leaflet side (51). Although the location of the functional binding site is still a matter of debate, we observed for the first time the direct binding of KcsA to PIPns as well as a tendency for the binding affinity to increase with the number of negative charges (Fig. 5). PI(3,4)P_2_, PI(4,5)P_2_, and PI(3,4,5)P_3_, which have three and four net negative charges, respectively, produced a stronger effect for the channel opening than POPG, which has one negative charge (Figs. 1*C*, 2, and 4). It is also worth noting that recordings from lipid membranes containing PI(3,4)P_2_, PI(4,5)P_2_, or PI(3,4,5)P_3_ showed a subconducting state and a large open channel noise (Fig. 3). It can be assumed that the presence of multiple negative charges causes instability in the interaction with their countercharges by charge swapping, resulting in fluctuation of the open permeation conformation; however, the details could not be explored. It has also been reported by molecular dynamics that interaction with lipids occurs when the activation gate opens at the C terminus of the second transmembrane helix (TM2) (52), and PIPns could be related to this mechanism. Recent NMR-based binding study indicated that including anionic lipids such as DOPG in proteoliposomes at acidic pH led to a weaker potassium ion affinity at the selectivity filter (51). The details of how the PIPns added to the inner leaflet in the present study affected the selectivity filter are not clear, but this idea may also explain the open channel noise. From another point of view, it is possible that the gating of KcsA was caused by the change in membrane tension due to the change in lipid composition caused by the addition of PIPns, as previously reported for membrane-stretch stimulation that changes the burst duration of KcsA activity (34). However, the high correlation with binding affinity (Fig. 6) suggests that the gating modification by PIPns is most likely a direct effect on the channel protein. As a similar phenomenon, the mechanosensitivity of *Mycobacterium tuberculosis* MscL (mechanosensitive channel of large conductance), a stretch-activated channel, is also enhanced when phosphatidylinositol is mixed into the lipid bilayer (53), and this mechanism may also be elucidated by the present analysis.

In this study, we analyzed the functional interaction of KcsA with PIPns and POPG. However, *S. lividans*, in which KcsA is expressed, does not have PIPns in its lipid membrane. In mammalian cells, on the other hand, PIPns is a major lipid responsible for the lipid signaling *via* functional modifications to ion channels. The significance of this study is that we were able to understand part of the molecular mechanism of functional modification by PIPns of large and complex mammalian K^+^ channels using small, simple, and tractable KcsA channels. In addition, ion channels (*e.g.*, Kir channels) have evolved to acquire specificity for different lipids, such as PIPns, and to exhibit unique physiological functions for each channel (54). In mammalian Kir and voltage-gated K^+^ channel (KCNQ), the binding of PI(4,5)P_2_ has been investigated regarding structural biology. In Kir, a binding pocket is formed in the region leading from the TM2 to the intracellular pore (25), and in KCNQ, a binding pocket is formed around the intracellular loop of the voltage-sensing domain (26). In both cases, the positively charged residues support the phosphate orientation. Interestingly, PI(4,5)P_2_ regulates Kir gating not only through a rigid binding pocket but also through electrostatic interactions with positively charged residues in the intracellular region (27). In fact, many mammalian ion channels have been reported to be modified by PIPns, either by a rigid structural basis, such as a binding pocket that recognizes the substrate, or by functional modification through ambiguous electrostatic interactions of substrate recognition (9, 23, 24). The present study using the KcsA K^+^ channel probably fits well into the latter category, and it is noteworthy that the functional modification of PIPns was observed in the reconstituted experimental system. Although the functional aspects of PI(4,5)P_2_-mediated regulation have been extensively analyzed using the inside-out patch-clamp method (17, 27), this is the first study to directly analyze intermolecular interactions and link them to ion channel function. PIPns are lipids found in the cytoplasmic leaflets and are responsible for signaling in biological membranes, with PI(4,5)P_2_ being the most abundant (about 1–3%) (1, 2, 3). In the present study, we found that even a concentration of 3% PI(4,5)P_2_ modified the activity of KcsA (Fig. 4). This suggests that the binding and functional modification mechanisms of PI(4,5)P_2_ identified here may also occur physiologically. In the future, it will be important to apply the method used in the present study to mammalian ion channels to clarify the modification mechanism of PIPns.

## Experimental procedures

### Reagents and chemicals

Lipids were purchased from Avanti Polar Lipids: 1-palmitoyl-2-oleoylphosphatidylcholine (POPC), 1-palmitoyl-2-oleoylphosphatidylglycerol (POPG), 1-palmitoyl-2-oleoyl-sn-glycero-3-phosphoinositol (POPI, PI), 1,2-dioleoyl-sn-glycero-3-phospho-(1′-myo-inositol-4′-phosphate) [PI(4)P], 1,2-dioleoyl-sn-glycero-3-phospho-(1′-myo-inositol-3′,4′-bisphosphate) [PI(3,4)P_2_], 1,2-dioleoyl-sn-glycero-3-phospho-(1′-myo-inositol-4′,5′-bisphosphate) [PI(4,5)P_2_], 1,2-dioleoyl-sn-glycero-3-phospho-(1′-myo-inositol-3′,4′,5′-trisphosphate) [PI(3,4,5)P_3_]. All other reagents and chemicals, such as for recording solutions and cell culture media, were purchased from FUJIFILM Wako Pure Chemical or Nacalai Tesque.

### Expression and purification of KcsA

The full-length KcsA gene (NCBI Accession No. P0A334) was synthesized by GENEWIZ and inserted into the pQE-82L vector (Qiagen) with a C-terminal 6 × His tag. The point mutation at Glu71 to Ala (E71A) in KcsA was generated using the PrimeSTAR Mutagenesis Basal Kit (Takara Bio).

KcsA was expressed in *Escherichia coli* BL21 (DE3) cells. Cells were grown in 2 × YT medium with ampicillin until absorbance at 600 nm (*A*_600_) = 0.5 to 0.6 at 37 °C. Then, protein expression was induced by the addition of 0.5 mM isopropyl-β-D-thiogalactopyranoside (IPTG) and followed by 2 to 3 h incubation. Cells were harvested and suspended in sonication buffer (20 mM Hepes [pH 7.2], 150 mM NaCl, 2 mM 2-mercaptoethanol, and Complete protease inhibitor cocktail tablets without EDTA) (Roche). Cells were lysed with an ultrasonic disruptor, and the membrane fractions were collected by ultracentrifugation (30,000 rpm, 1 h). A solubilization buffer (20 mM KPi pH 7.5, 200 mM KCl, 10 mM imidazole, 2 mM 2-mercaptoethanol, and 1.0% n-dodecyl-β-D-maltoside [DDM]) was then added, and cells were incubated at room temperature for 1 h. The supernatants were loaded onto Co^2+^-based affinity resin (TALON Metal Affinity Resin, Takara Bio), and His-tagged KcsA proteins were eluted with 200 mM imidazole. The eluted proteins were concentrated by ultrafiltration (Amicon Ultra-0.5, Merck KGaA). Protein purity was checked by SDS-PAGE and Coomassie brilliant blue staining.

Purified channel proteins were reconstituted into liposomes using the following method. Stock lipids in chloroform were dried in a glass tube under a stream of nitrogen gas followed by vacuum overnight. Dried POPC lipid films were suspended in reconstitution buffer (20 mM Hepes, 200 mM KCl, pH 7.0) at a concentration of 2 mg/ml and bath-sonicated. Purified channels were reconstituted into the POPC liposome solution (protein:lipid = 1:1000, weight ratio) by at least 50 times dilution and incubated for 30 min at room temperature. Proteoliposomes were stored at −80 °C until use. To form asymmetric lipid bilayers, empty liposomes consisting of various lipid compositions without proteins were prepared using the same procedure, except for the lipid suspension buffer (20 mM citrate, 200 mM KCl, pH 4.0).

### Electrophysiology

The CBB method (39, 40) was used for all recordings. Borosilicate capillary glass pipettes (Calibrated Pipet 75 μl; Drummond Scientific) were used for bubble formation through a micropipette puller (P-97; Sutter Instrument). The tip of the pipette was broken and slightly polished using a microforge (MF-830; Narishige). The pipettes were attached to pipette holders and operated by a motor-driven micromanipulator (MP-225; Sutter Instrument) and a manual micromanipulator (NMN-21; Narishige) on an inverted microscope (IX-71; Olympus). The pressure in the pipettes was regulated by a pneumatically operated microinjector (IM-11-2; Narishige). A few microliters of liposome solutions with or without proteins (2 mg/ml) were added to the tips of the glass pipettes by applying negative pressure. Two water bubbles were formed on both sides of the glass pipettes by applying positive pressure inside the pipettes in the oil phase (hexadecane) and maintained for a few minutes to stabilize the bubbles. The bubbles contacted each other by pipette manipulation to form the bilayer at the interface in the center.

Single-channel currents were recorded using an Axopatch 200B amplifier (Molecular Devices). The extracellular-side solution contained 200 mM KCl and 20 mM Hepes (pH 7.0), and the intracellular-side solution contained 200 mM KCl and 20 mM citric acid (pH 4.0). Stimulation, data acquisition, and analysis were performed on a computer using a Digidata 1440A AD/DA converter and pClamp 10.3 or 10.7 (Molecular Devices). The recorded currents were low-pass filtered at 5 kHz using a four-pole Bessel filter circuit built in the amplifier, and the sampling frequency was 10 to 20 kHz. The current traces were digitally filtered at 1 kHz. Recordings were performed at room temperature. CBB measurements are subject to variations in measurement time because of the limited time that bubbles are in contact with each other, *i.e.*, the time during which the bilayer is formed. Multiple measurement trials were performed for each lipid, but simply addition-averaging the Po of each trial did not yield an accurate Po because of the different recording times (5 s to 271 s). To calculate a more accurate Po, we calculated Po for every 1 s segment of all current recordings; all Po values for every 1 s are plotted and presented as mean ± standard deviation (SD) (Figs. 2 and 4). This method allowed us to analyze the effect of each lipid on Po with less measurement bias. The number of the data segments and the measurement trials for each lipid were listed in the figure legends (Figs. 2 and 4). The total number of events that crossed the threshold during single-channel analysis were 4473 for 100% POPC, 13,553 for 10% POPG, 1254 for 10% PI, 2342 for 10% PI(4)P, 78,371 for 10% PI(3,4)P_2_, 28,651 for PI(4,5)P_2_, and 7889 for 10% PI(3,4,5)P_3_, which were sufficient for analysis (Fig. 2). The single-channel amplitude was estimated from the peak-to-peak amplitude of the event histogram with a bin width of 0.5 pA (Fig. 2). Open channel noise was estimated using the root-mean-squares of the intraburst current amplitude during a measurement trial (Fig. 3). All data obtained in the measurements are plotted and shown as mean ± SD (Figs. 2*B*, 3*B*, and 4*C*). Data were examined for significant differences between multiple groups by one-way ANOVA with Tukey–Kramer test (Figs. 2 and 3). Statistical tests between the two groups were performed using the Student’s *t* test (Fig. 4). Data were analyzed using Excel (Microsoft), SPSS (IBM), Clampfit (Molecular Devices), and Igor Pro (WaveMetrics) software.

### Protein–lipid interaction analysis

The MST analysis for the interaction between KcsA and lipids was performed using Monolith NT.115 (NanoTemper Technologies GmbH), following the standard assay protocols (49, 50). KcsA was labeled using the Monolith His-Tag Labeling Kit RED-tris-NTA 2nd Generation (NanoTemper Technologies GmbH) according to the manufacturer’s protocol. KcsA proteins and lipids were dissolved in the MST analysis buffer containing 150 mM KCl, 10 mM Hepes (pH 7.0, adjusted with NaOH), and 0.1% Tween 20. The detergent was changed from DDM to Tween to prevent aggregation, in which concentrated 25 μM KcsA protein dissolved in buffer containing 0.1% DDM was diluted 500-fold with the MST analysis buffer to dilute DDM to below the critical micelle concentration. KcsA was used as a binding target for MST analysis at a concentration of 50 nM. Each lipid tested as a ligand in the MST analysis was titrated into the KcsA solution in a 1:1 dilution series over 0.25 to 0.0000153 mM. KcsA and diluted lipid samples were mixed and loaded into a Monolith premium capillary (NanoTemper Technologies GmbH), and the MST fluorescence was measured at 25 °C using the MO.Control software bundled with Monolith NT.115, in which the excitation power and the MST power were both set at 40%. Data were analyzed using the MO.Affinity Analysis software (version 3.0.4, NanoTemper Technologies GmbH) under standard and default MST-on time conditions (4–5 s: gray area in Fig. 5*A*). F_norm_ ‰ is the thousandth fraction of the normalized value of MST fluorescence before thermophoresis as 1. Ligand-dependent photobleaching was detected at a high concentration range (approximately 0.25–0.625 mM) in all lipids, and there data were excluded from the analysis. K_d_ was estimated by fitting the data with the following equation in the software:

(1) $$f\left(c\right)=F_{unbound}+\left(F_{\text{bound}}-F_{\text{unbound}}\right)\cdot \frac{c+c_{\text{target}}+K_{d}-\sqrt{{\left(c-c_{\text{target}}+K_{d}\right)}^{2}-4\cdot c\cdot c_{\text{target}}}}{2\cdot c_{\text{target}}},$$

where f(c) is the fraction bound at a given ligand concentration c, F_unbound_ is the F_norm_ signal of the target alone, F_bound_ is the F_norm_ signal of the complex, and C_target_ is the final concentration of the target in the assay. K_d_ values were log-transformed and subjected to the one-way ANOVA with Tukey–Kramer test to examine significant differences between multiple groups. All K_d_ values obtained in the measurements are plotted and shown as mean ± SD (Fig. 5*C*). In Figure 6, K_d_ values were log-transformed and correlations with Po were analyzed by linear regression. Data were statistically analyzed using Excel (Microsoft) and SPSS (IBM) software and plotted using Igor Pro software (WaveMetrics).

## Data availability

The authors confirm that the data supporting the findings of this study are available within the article.

## Conflict of interest

The authors declare that they have no conflicts of interest with the contents of this article.
